# Nature-inspired spider web shaped UHF RFID reader antenna for IoT and healthcare applications

**DOI:** 10.1038/s41598-023-39825-9

**Published:** 2023-08-28

**Authors:** Abubakar Sharif, Rajesh kumar, Kamran Arshad, Khaled Assaleh, Hassan Tariq Chattha, Muhammad Ali Imran, Qammer Hussain Abbasi

**Affiliations:** 1 Yangtze Delta Region Institute (Huzhou), University of Electronic Science and Technology of China (UESTC), Huzhou, China; 2 Department of Electrical Engineering and Technology, Government College University Faisalabad (GCUF), Faisalabad, Pakistan; 3 Department of Electrical and Computer Engineering, College of Engineering and Information Technology, Ajman University, Ajman, United Arab Emirates; 4 Artificial Intelligence Research Center (AIRC), Ajman University, Ajman, United Arab Emirates; 5 Advanced Cyclotron Systems Inc. (ACSI), Richmond, BC V6X 1X5 Canada; 6 James Watt School of Engineering, University of Glasgow, Glasgow, G12 8QQ UK

**Keywords:** Electrical and electronic engineering, Biomedical engineering

## Abstract

This paper proposes a nature-inspired spider web-shaped ultra-high frequency (UHF) radio frequency identification (RFID) reader antenna and battery-free sensor-based system for healthcare applications. This antenna design consists of eight concentric decagons of various sizes and five straight microstrip lines.These lines are connected to the ground using 50 $\Omega$ resistors from both ends, except for one microstrip line that is reserved for connecting a feeding port. The reader antenna design features fairly strong and uniform electric and magnetic field characteristics. It also exhibits wideband characteristics, covering whole UHF RFID band (860–960 MHz) and providing a tag reading volume of 200 $\times$ 200 $\times$ 20 mm${^3}$. Additionally, it has low gain characteristics, which are necessary for the majority of nearfield applications to prevent the misreading of other tags. Moreover, the current distribution in this design is symmetric throughout the structure, effectively resolving orientation sensitivity issues commonly encountered in low-cost linearly polarized tag antennas. The measurement results show that the reader antenna can read medicine pills tagged using low-cost passive/battery-free RFID tags, tagged expensive jewelry, intervenes solution, and blood bags positioned in various orientations. As a result, the proposed reader antenna-based system is a strong contender for near-field RFID, healthcare, and IoT applications.

## Introduction

Radio frequency identification (RFID) is being combined with the Internet of Things (IoT) to predict exciting applications^1–6^. The UHF RFID system has the advantages of long reading range, many tags reading capabilities, simple tag structure, fast reading speed, etc., and has broad application prospects^7^. Near-field RFID tag systems are more suitable for item-level tags and environmentally sensitive applications. It is necessary to successfully read valuables goods with 100 % probability such as medicines, and jewelry. However, one of the key technical issues in the design of UHF near-field RFID reader antennas is to generate a sufficiently strong and relatively uniform field distribution^8–15^. In addition, low-cost commercial tag antennas are linearly polarized posing orientation sensitivity issues. Therefore, there is a need for a reader antenna with uniform magnetic and electric field characteristics. In addition to this, the near-field reader antenna that solves tag’s orientation sensitivity issues will be more advantageous.

In^16^, a broadband RFID reader antenna with low far-field gain has been devised for near-field applications. The antenna was made up of several loop units, each of which has an in-phase current running through the loop. It produces a powerful magnetic field that is evenly distributed over a large interrogation zone. The interrogated area can be modified as a result of altering the number of units to accommodate various scenarios. Low far-field gain and broadband characteristics are features of the suggested antenna. However, this antenna has large dimensions 776 mm $\times$ 120 mm $\times$ 1 mm.

A UHF RFID reader antenna based on two microstrip meander lines with open ends. The equivalent currents in two meander lines are reversed in phase and have almost equal amplitudes. Meander-line units were used to create a homogeneous distribution of both magnetic and electric fields. The performance of the proposed reader antenna was analyzed using six pairs of meander lines. This reader antenna achieved small bandwidth of 914 to 929 MHz with a large footprint of 480 mm $\times$ 200 mm $\times$ 1.6 mm^17^. A near-field RFID reader antenna based on a meander line structure has been proposed in^18^. This antenna design was composed of one open-ended microstrip meander line structure and another meandered line with 50 $\Omega$ termination coupled together to get a fairly strong electric field in the near-field region.

The antenna dimensions are 140 $\times$ 100 $\times$ 2 mm${^3}$ with multi tags reading volume of 170 $\times$ 150 $\times$ 25 mm${^3}$. Another complementary split ring resonator (CSRR) based near field reader antenna has been designed in^19^. The antenna was based on a power divider and two 50 $\Omega$ terminated microstrip arms. One arm is loaded with CSRR elements, activating backward wave propagation. The reader antenna achieved 0.76 to 0.88 GHz with an interrogation plane of 220 mm $\times$ 180 mm $\times$ 50 mm. In^20^, a wearable Yagi-like near-field reader antenna has been investigated to be integrated into a smart glove together with s small RFID reader. This design is based on a rhombus-shaped folded dipole and parasitic elements (acting as either a reflectors or directors). Different variants of this Yagi antenna were designed on stretchable fabric and their performance is analyzed in terms of near-field distribution and reflection coefficient. A multi-polarized near-field RFID reader antenna based on two symmetrical meandered and open-ended microstrip lines was proposed in^19^. The 90o phase shift in one of microstrip line achieved multi-polarization and a strong electric field in the near-field region. This reader antenna has a reading volume of 450 mm $\times$ 450 mm $\times$ 350 mm with 255.6 mm $\times$ 220 mm $\times$ 1 mm dimensions. A multi-polarized nearfield UHF RFID reader with periodic units has been proposed for near-field applications based on electric field coupling^21^. Most of the aforementioned near-field reader antennas were based on meandered line structures that are quite large and have small bandwidth.

**Figure 1:**
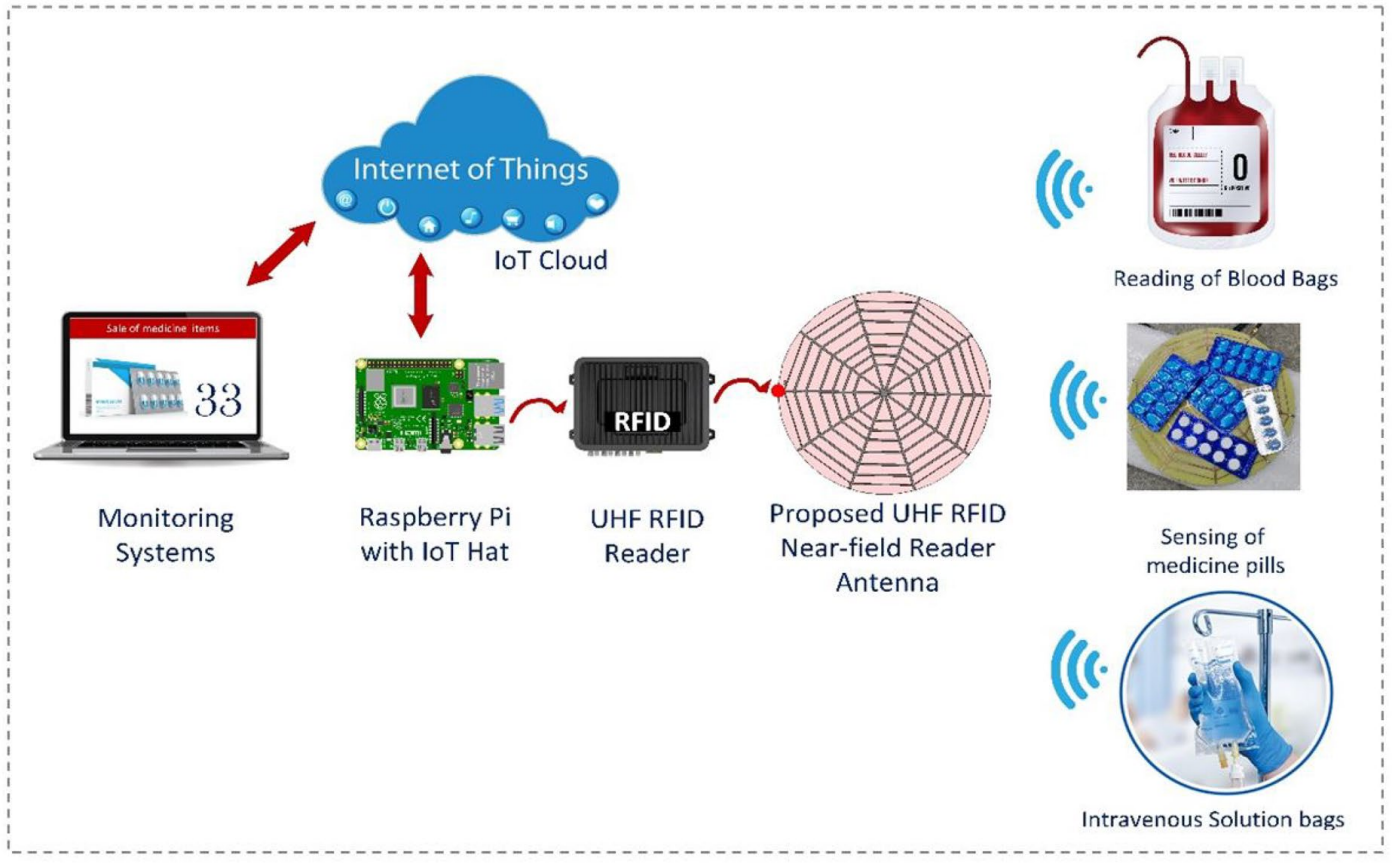
Geometry and structure of proposed UHF RFID Near field reader Antenna with detailed dimensions (all dimensions are in mm).

Therefore, in this paper, a spider web-shaped ultra-high frequency (UHF) RFID reader antenna for the Internet of Things (IoT) and healthcare applications is proposed. The proposed reader antenna is composed of eight concentric decagons of various sizes and five straight microstrip lines. These lines are connected to the ground from both ends using 50 $\Omega$ resistors, except for the end left open for a feeding port. The reader antenna design has wideband properties because it covers the entire UHF RFID band (860–960 MHz) and has fairly strong and uniform electric and magnetic field characteristics. Moreover, this antenna poses low gain characteristics, which are necessary for the majority of nearfield applications in order to avoid misreading other tags. Additionally, the symmetric current distribution of this structure addresses the orientation sensitivity problems with a low-cost linearly polarized tag. The test results demonstrate that the reader antenna can read tags on expensive jewelry, blood bags, intervention solution, and pill bottles that have been tagged with inexpensive passive/battery-free RFID tags. The suggested reader antenna-based system is a robust contender for near-field healthcare, and IoT applications as illustrated in Fig. 1.

## Concept and antenna design

**Figure 2:**
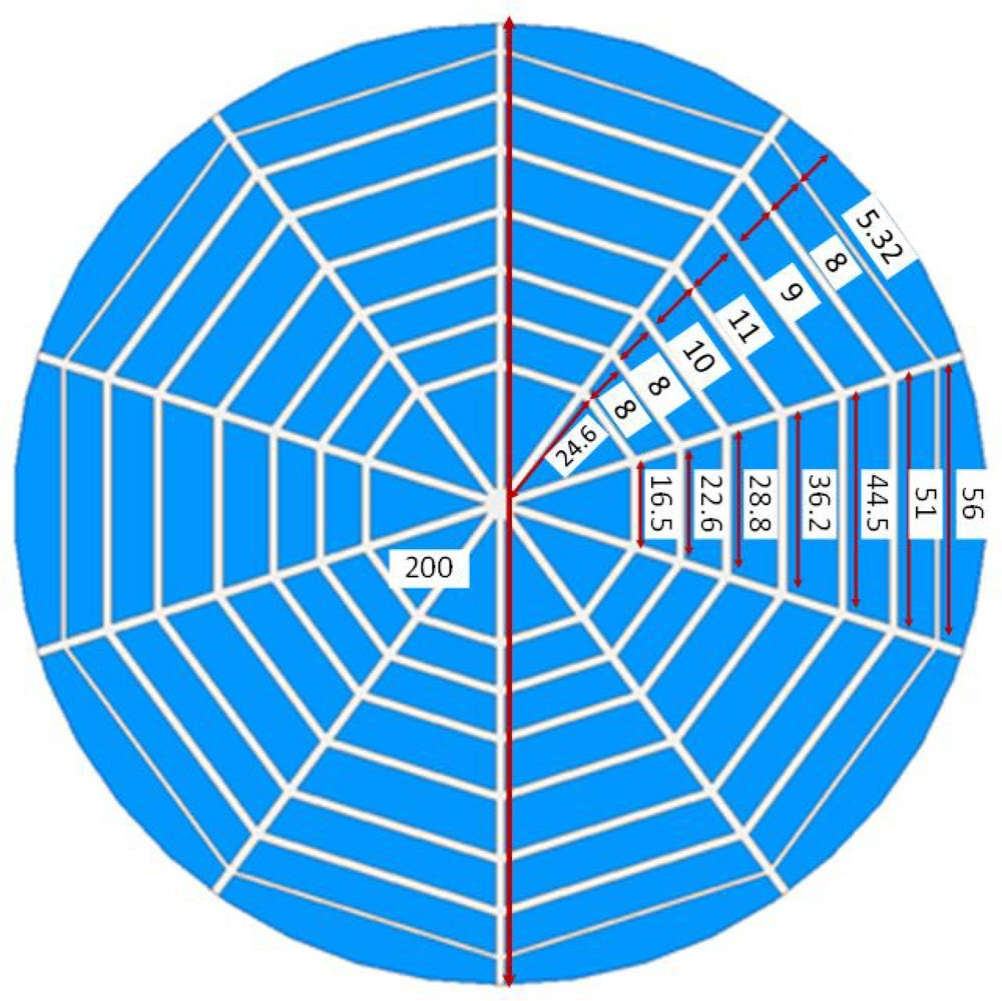
Geometry and structure of proposed UHF RFID Near field reader Antenna with detailed dimensions (all dimensions are in mm).

Figure 2 shows the geometry and structure of the proposed UHF RFID Near field reader Antenna with detailed dimensions. The proposed tag antenna consists of a spider web-shaped structure etched on a low-cost RF-4 substrate ($\epsilon r=4.4, \tan \delta =0.02)$) with a ground plane. The proposed design integrates seven concentric decagons and five open-ended microstrip lines that are fabricated on a circular FR4 substrate with a radius of 100 mm and a thickness of 1.5 mm. CST Microwave Studio is used to design and optimize the antenna. For all decagon and microstrip lines, the copper trace width is 2 mm except for the last decagon, whose width is optimized to 1 mm.

**Figure 3:**
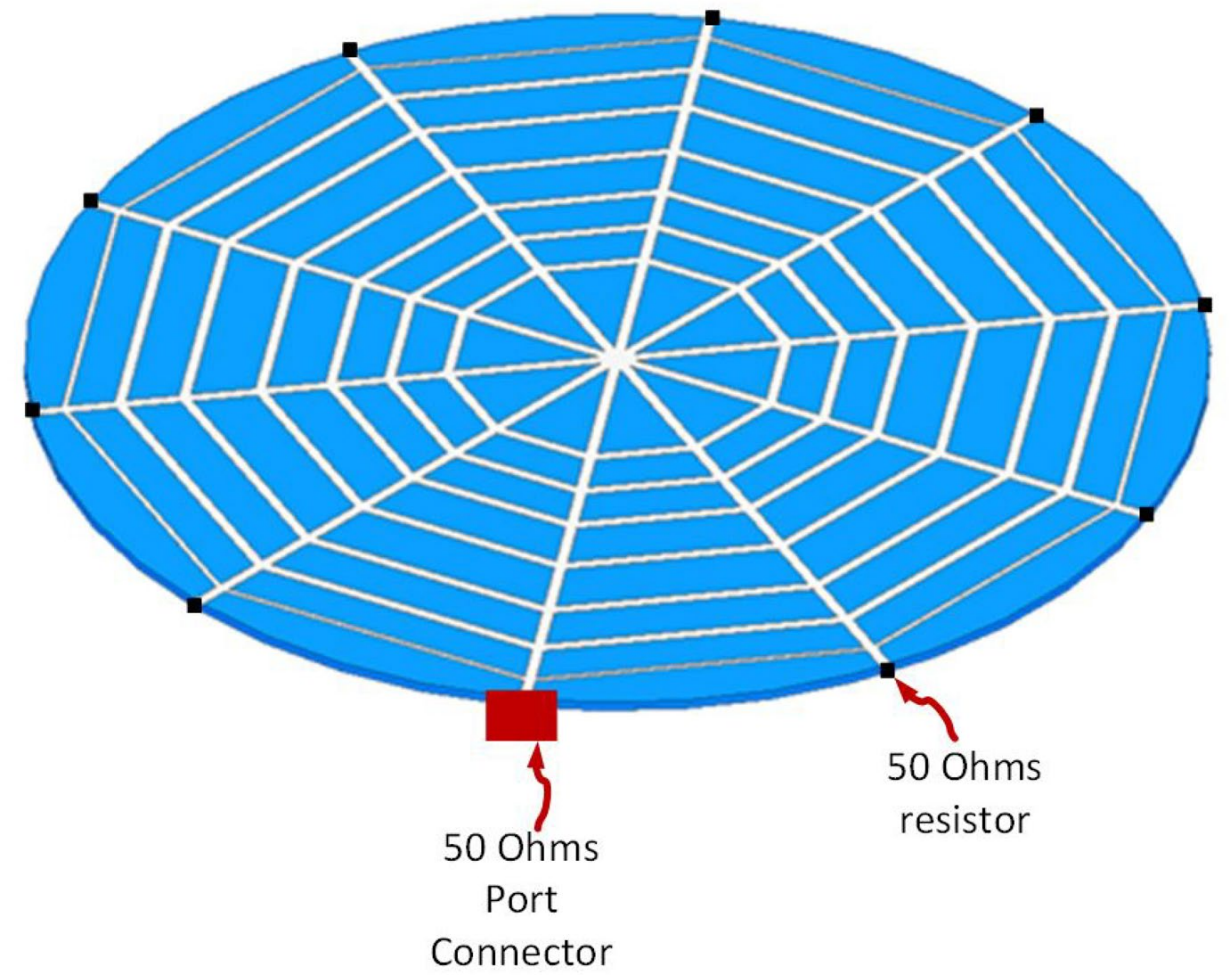
Side view of proposed reader antenna with 50 resistor terminations.

Figure 3 shows a complete reader antenna configuration with 50 $\Omega$ termination at both ends of straight microstrip lines. One end of single microstrip is left open for open for a feeding port. Due to the symmetric structure of the proposed spider web antenna, the port can be attached to any single end of a straight microstrip line. The 50 $\Omega$ resistors are connected vertically between the ends of the microstrip line and ground plane.

## Simulated and measured results

**Figure 4:**
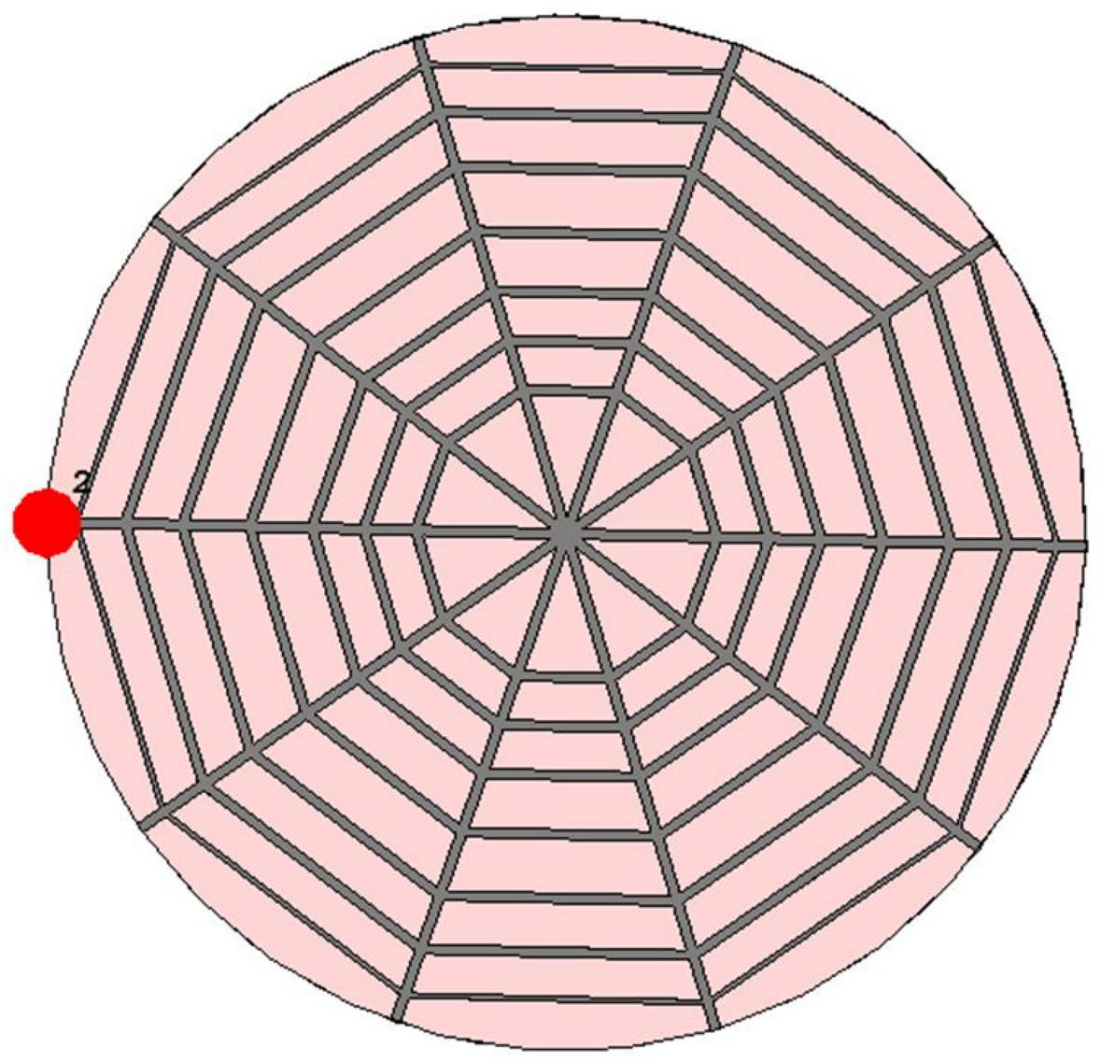
Initial design of spider-web near-field reader antenna.

**Figure 5:**
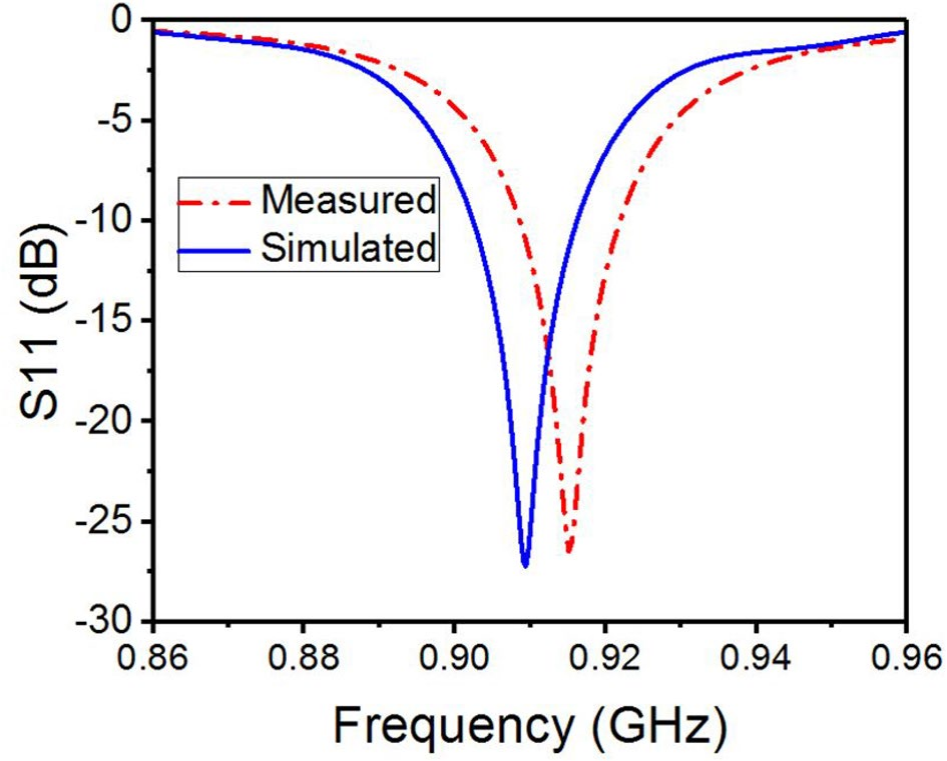
Simulated and measured S11 parameter of initial spider-web antenna design.

**Figure 6:**
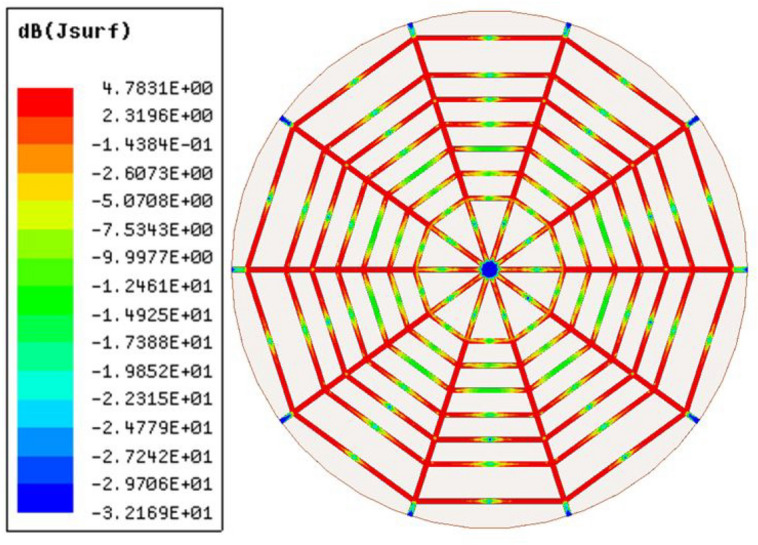
The surface current distribution of initial design of spider-web near-field reader antenna.

Figure 4 illustrates the initial design of the spider-web near-field reader antenna. The initial design is fed from one straight microstrip line, while all other microstrip lines are left open-ended. The corresponding simulated and measured S11 parameter of this initial design is depicted in Fig. 5. The S11 shows a bandwidth ranging from 900 to 920 MHz. There is a little discrepancy in measured S11, which may be due to the difference in the dielectric constant of simulated and fabricated FR-4 substrates. Similarly, the surface current distribution of the initial spider-web antenna design is shown in Fig. 6.

Figure 6 shows a symmetric current distribution of the initial design, however, this current becomes zero at the ends due to the open-ended structure. The initial design also solves tag orientation issues due to current symmetry. However, the bandwidth, electric and magnetic field parameters need some improvement.

**Figure 7:**
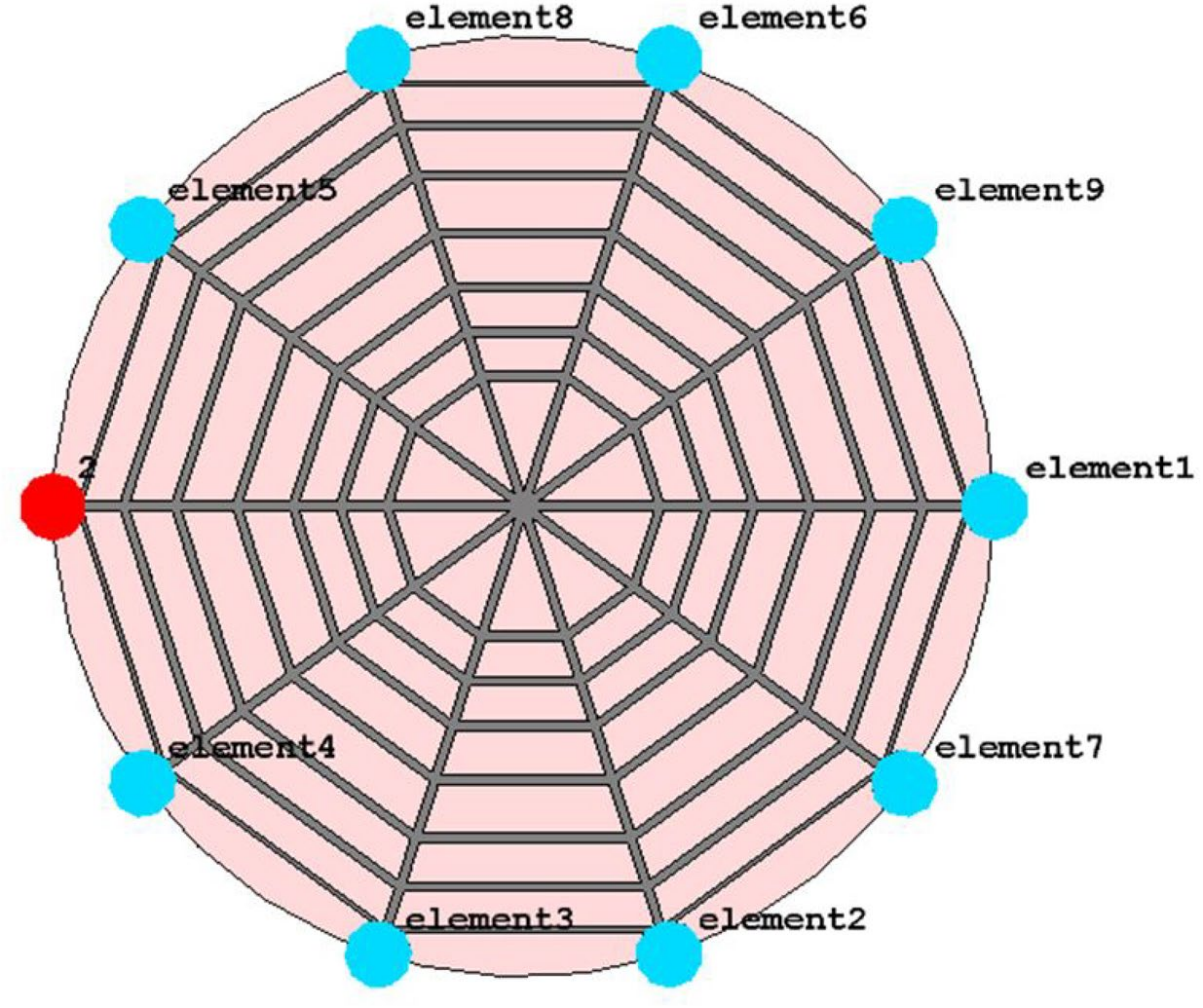
Final configuration of proposed spider-web near-field reader antenna.

Therefore, the initial structure is optimized further using CST Microwave Studio by applying resistive loading at open ends of straight microstrip lines as illustrated in Fig. 7. The Final configuration of the proposed spider-web near-field reader antenna with feed port and 50 $\Omega$ match terminations is depicted in Fig. 7. The 50 $\Omega$ resistive loading balances out the surface current in the proposed spider-web reader antenna.

**Figure 8:**
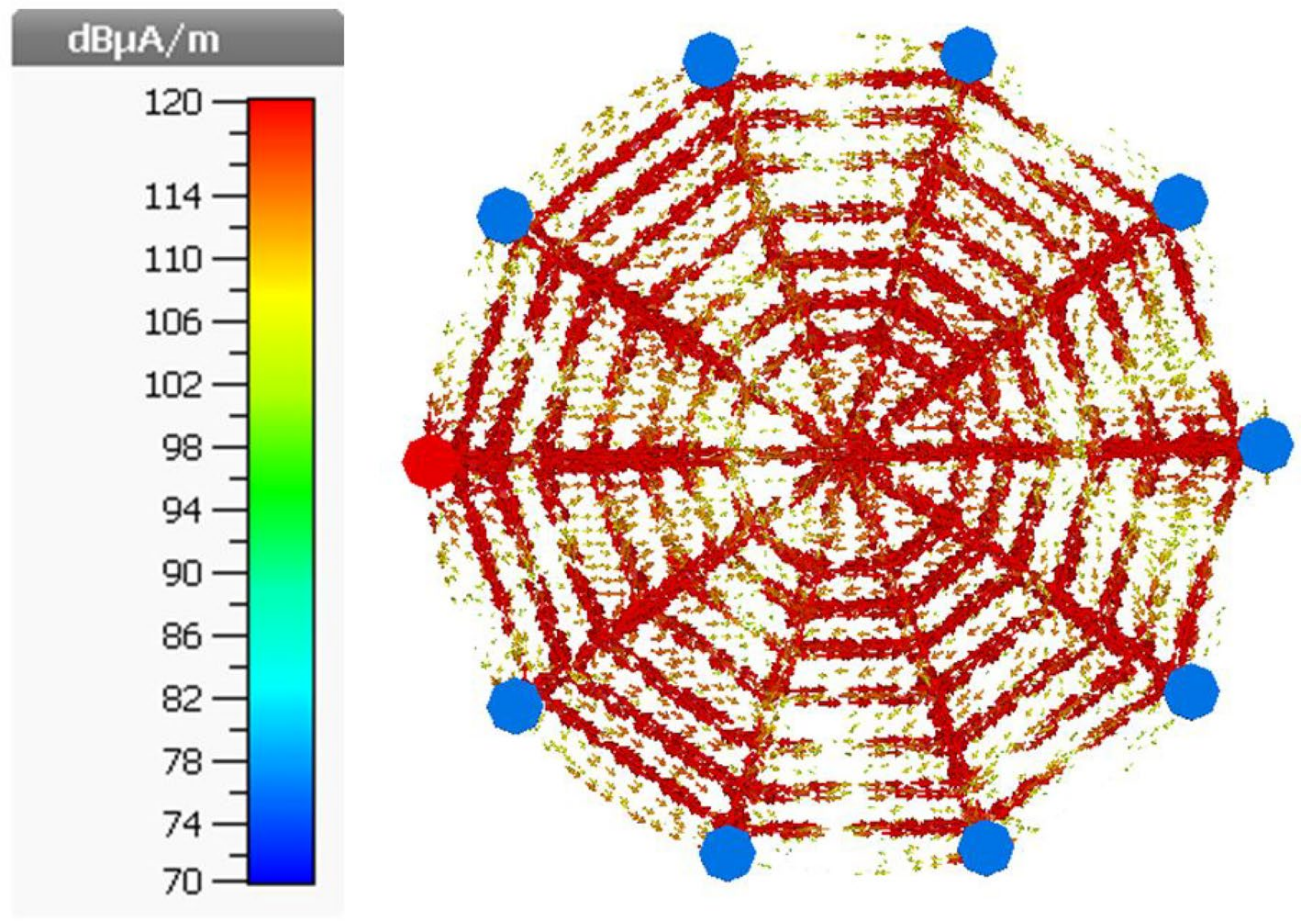
Surface current distribution of Final Proposed spider-web near-field reader antenna.

Figure 8 shows the Surface current distribution of the final proposed spider-web near-field reader antenna. The 50 $\Omega$ matched termination diminishes the current reflections at the edges of straight microstrip lines. Consequently, the surface current looks more fairly distributed and symmetric in this final reader antenna configuration.

**Figure 9:**
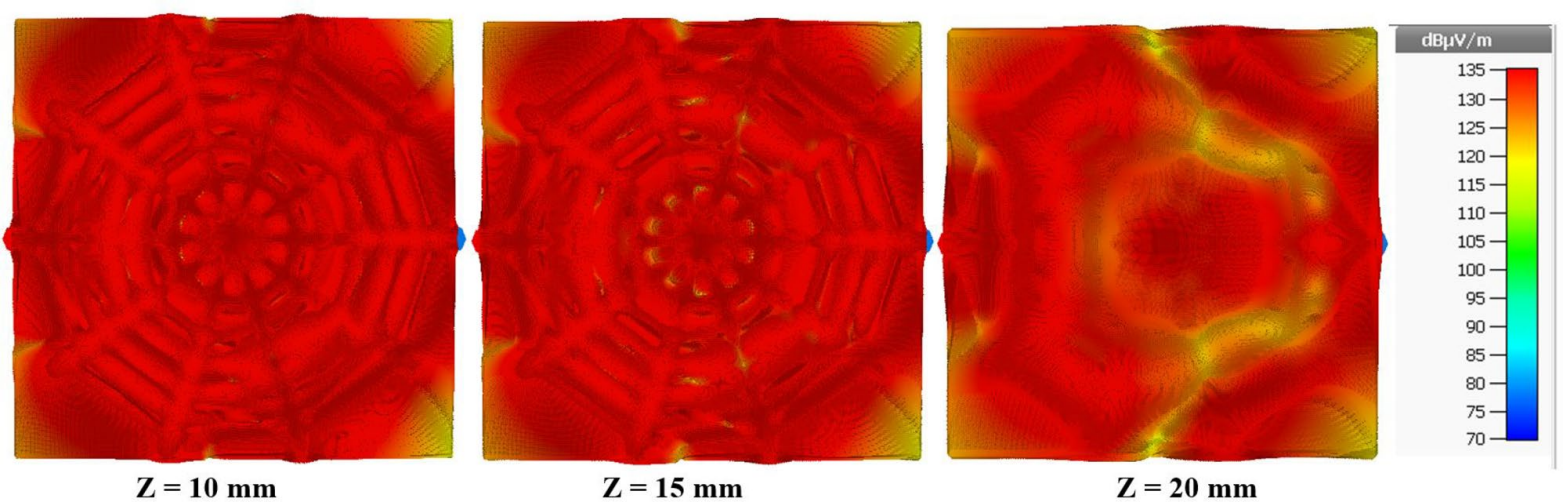
Simulated electric field distribution of proposed reader antenna at 915 MHz.

Figure 9 illustrates the electric field distribution in xy-plane at z = 5 mm, z = 10 mm, z = 15 mm and z = 20 mm for final spider-web shaped reader antenna configuration at 915 MHz. It is clear that the reading zone denoted by the rectangular shapes superimposed inside the plots has a uniform distribution of the electric field intensities produced by the antenna. Moreover, the electric field intensities decrease as the reading height perpendicular to the antenna rises. As mentioned in^15^, If the electric field distribution is greater than 85 dB (1 mV/m), the reader antenna can read all tags simultaneously. As a result, the electric field distribution range was set to be between 70 and 90 dB. Furthermore, this reader antenna achieved a tag reading volume reading region of 200 mm $\times$ 200 mm $\times$ 25 mm with uniform electric field distribution.

**Figure 10:**
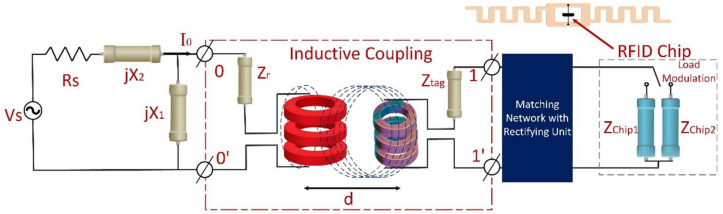
Circuit model for near-field magnetic field coupling of UHF RFID systems.

Furthermore, a circuit model for near-field magnetic field coupling of UHF RFID systems is discussed in order to understand the magnetic field distribution of the proposed spider-web reader antenna. The UHF RFID near-field technology utilized inductive coupling principles similar to transformer coupling^18,22^ as depicted in Fig. 10. The reader and tag antennas can be mimicked as primary and secondary coils, respectively. The near-field UHF RFID systems pose more reading distance of more than a dozen centimeters as compared to other traditional near-field systems, where the distance is less than 0:16 $\lambda$ (about 5 cm).

As referred to circuit model, Rs and Zr are the reader’s power supply resistance (which is usually 50 $\omega$) and impedance of reader antenna, respectively. X1, and X2, are the reactive components associated with an impedance of reader antenna. While Ztag is the impedance of tag antenna. If the reader antenna matching coefficient is equal to 1, and Rs-jX2 is equal to *Zr*/*jX*1, the current I0 and coupling power can be represented as follows^23^:

1 $$\begin{aligned}{} & {} I_0 \approx \sqrt{\frac{R_s}{R_r}} \frac{V_s}{2 R_s}\left( 1+\frac{(w M)}{4 R_r R_{\text{ tag } }}\right) ^{-1} \end{aligned}$$

2 $$\begin{aligned}{} & {} P_{\text{ Coupling } } \approx \frac{1}{8 R_{\text{ tag } }} \cdot \left| j w M \sqrt{\frac{R_s}{R_r}} \frac{V_s}{2 R_s}\left( 1+\frac{(w M)}{4 R_r R_{\text{ tag } }}\right) ^{-1}\right| ^2 \end{aligned}$$

whereas $P_{coupling}$ power delivered to the tag’s chip, M is mutual coupling coefficient between reader and tag antennas. $Zr = Rr+ jwMLr$ and $Ztag = Rtag+ jwMLtag$ are reader antenna and tag impedances.

**Figure 11:**
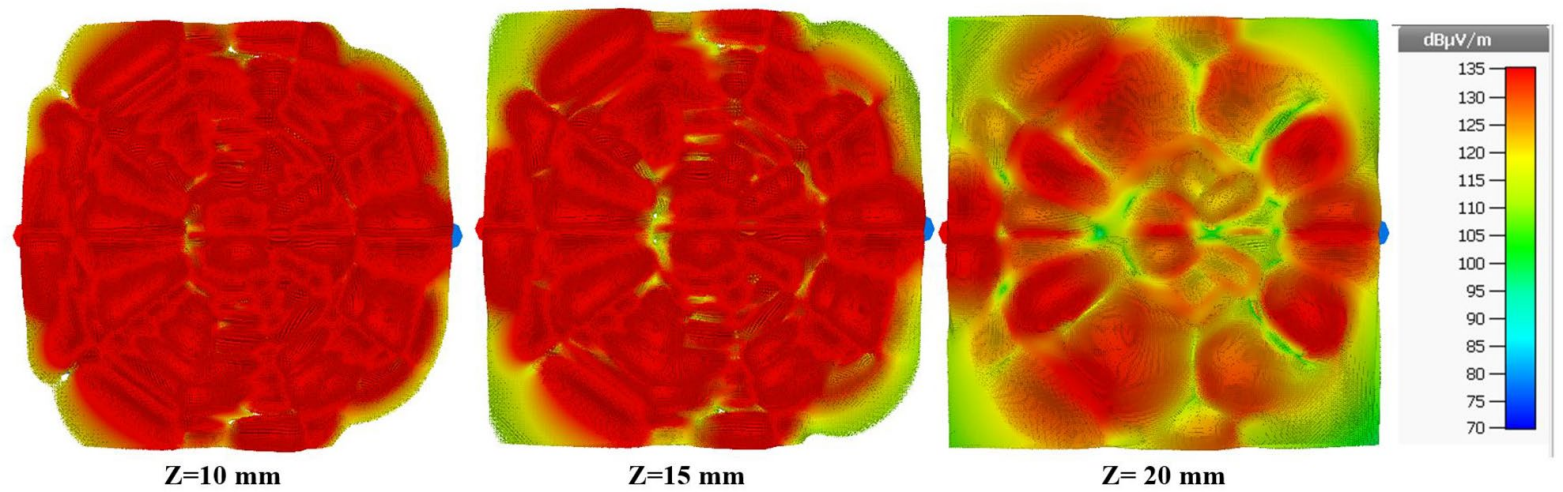
Simulated magnetic field distribution of proposed reader antenna at 915 MHz.

Therefore, the simulated magnetic field distribution of the proposed spider-web reader antenna at 915 MHz is illustrated in Fig. 11. The magnetic field distribution is plotted in xy-plane at z = 5 mm, z = 10 mm, z = 15 mm and z = 20 mm. The combination of decagons and straight microstrp lines terminated with matched load produces an electromagnetic coupling mechanism that can able to detect/read UHF RFID tags in a near-field zone. It is clear that the reading zone denoted by the rectangular shapes superimposed inside the plots has a uniform distribution of the magnetic field intensities produced by the antenna.

This proposed reader antenna also accomplished a tag reading volume reading region of 200 mm $\times$ 200 mm $\times$ 20 mm with uniform magnetic field distribution. Moreover, the uniformity of magnetic field intensity suddenly drops after z = 25 mm.

**Figure 12:**
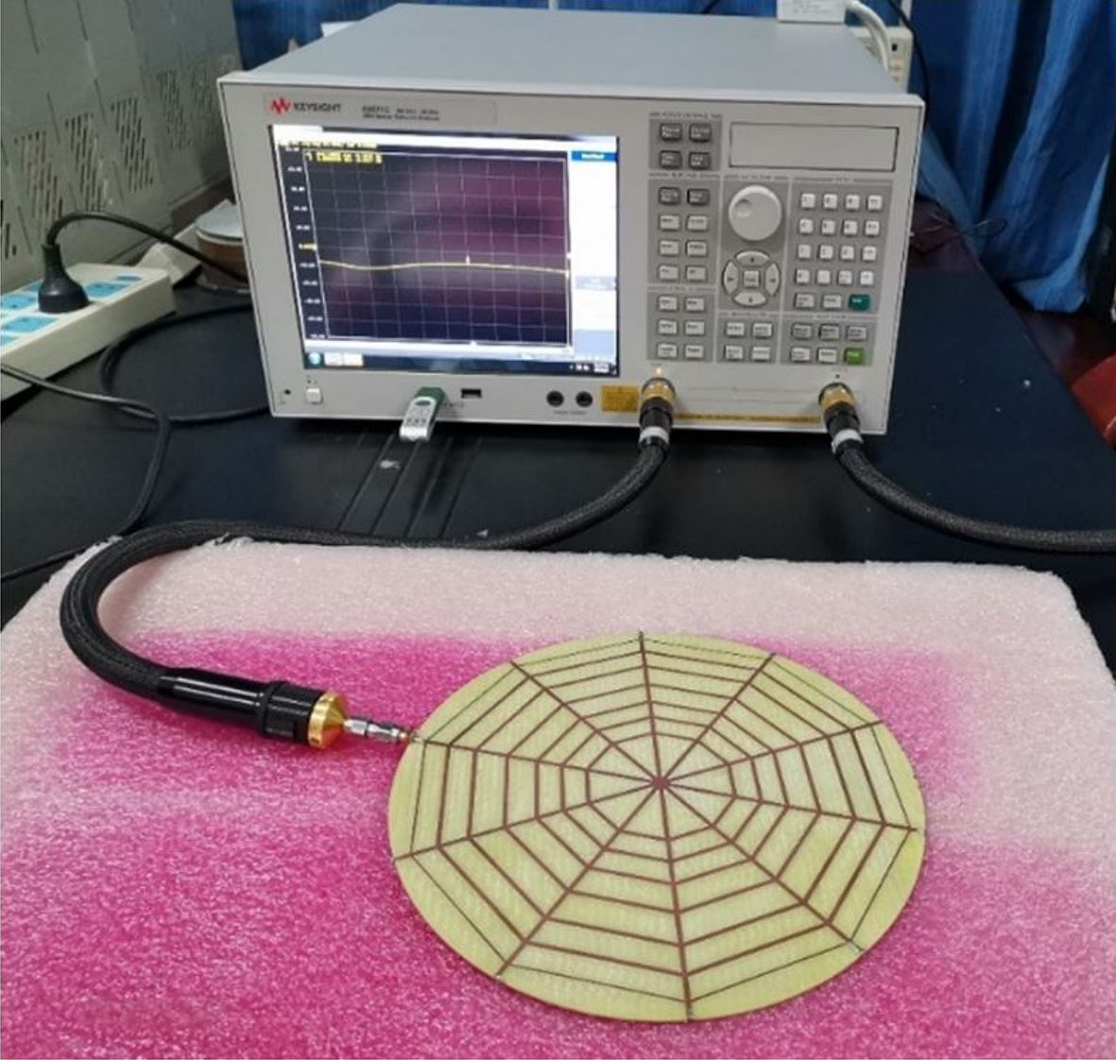
S11 parameter measurement setup for spider-web reader antenna.

Figure 12 shows a fabricated porotype of a spider-web reader antenna with feeding port and 50 $\omega$ resistors etched on FR-4 substrate. This fabricated antenna is used for S11 and tag read testing proposes. The S11 measurement setup for the spider-web reader antenna is also illustrated in Fig. 12. The S11 parameter is measured using the Agilent E8363B vector network analyzer (VNA).

**Figure 13:**
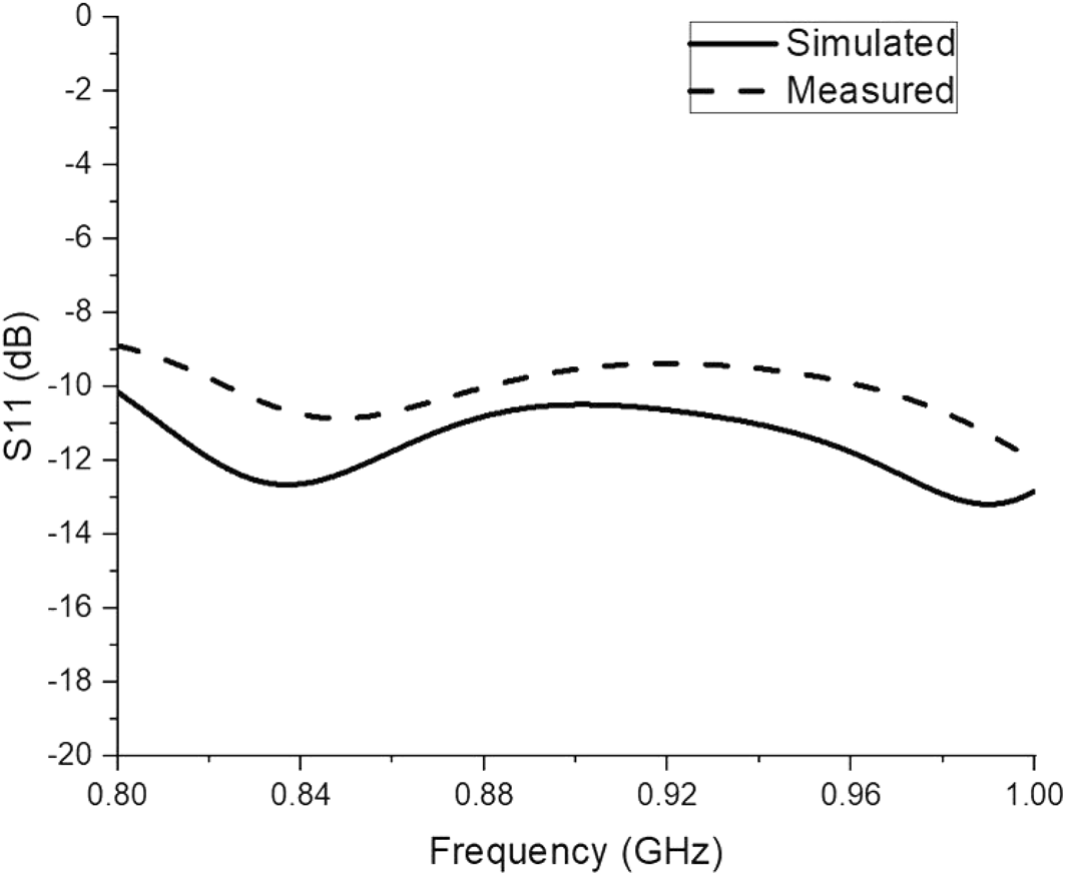
Measured and simulated S11 of proposed spider-web antenna.

Figure 13 shows the measured and simulated S11 parameter of the proposed spider-web reader antenna. It can be observed that the reader antenna features a wide bandwidth ranging from 800 to 1000 MHz, and covers the whole UHF RFID band (860–960 MHz). Recalling Fig. 5, the S11 parameter of the initial version with an open-ended straight microstrip line has a very narrow bandwidth (900–920 MHz) as compared to the final spider antenna with 50 $\Omega$ matching terminations.

In fact, the matched termination diminishes current reflections at open ends of straight microstrip lines, thereby contributing towards increasing the bandwidth of the proposed spider-web reader antenna. Moreover, there is a little discrepancy in simulated and measured results of the S11 parameter which may be due to the difference in the simulated and actual dielectric constant of the FR-substrate.

## Bio-sensing applications using proposed system

**Figure 14:**
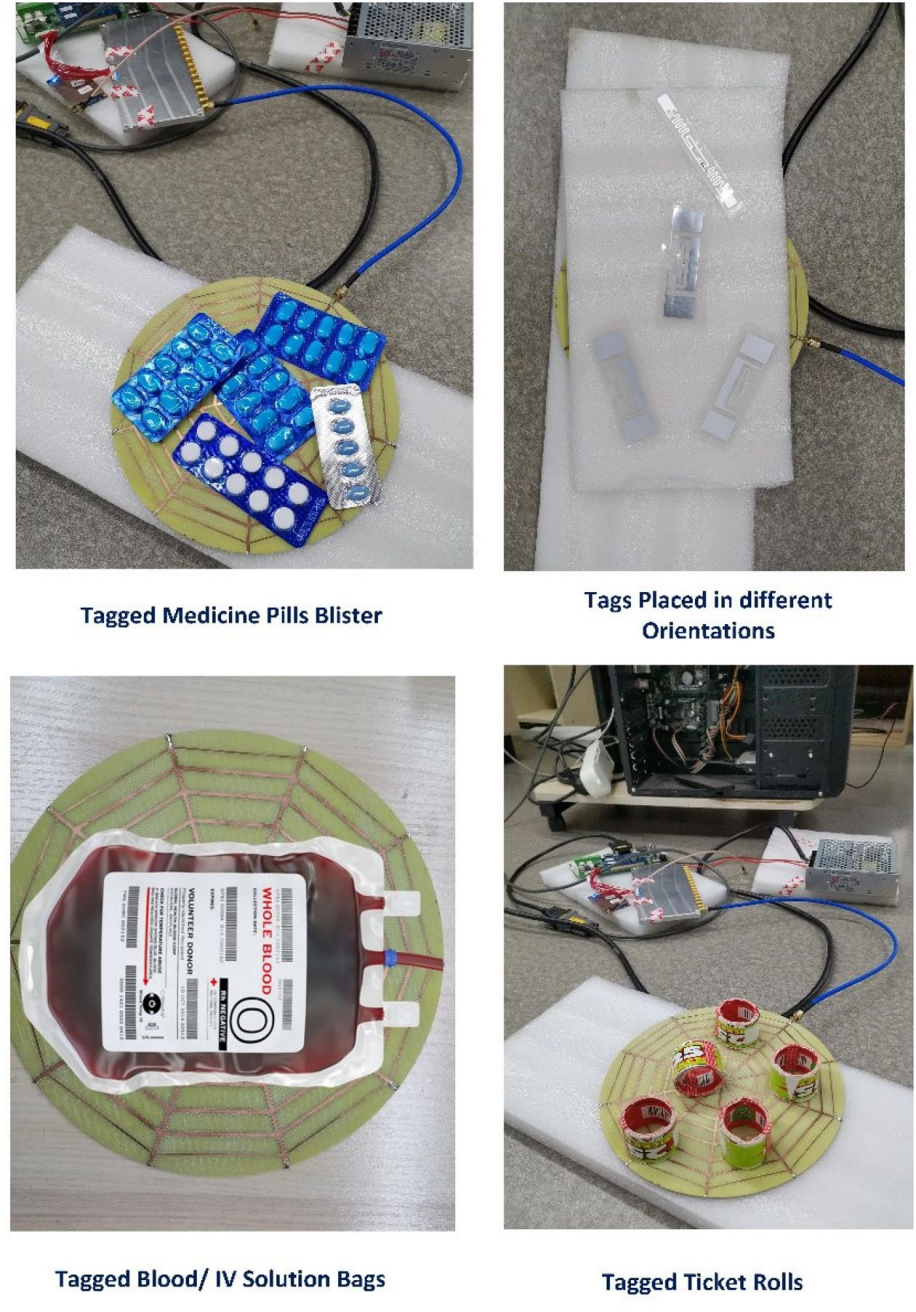
Applications of proposed spider-web reader antenna-based system for biomedical sensing and other item-level tagging scenarios.

As illustrated in Fig. 14, the fabricated antenna was connected to an Impinj reader setup with 30 dBm output power for tag reading experiments. The left inset of Fig. 14 shows, that the reader antenna can read tagged medicine pills blisters that were positioned above 10 mm foam in various orientations. Moreover, this reader antenna can successfully read all tagged medicine blisters with a 100% reading probability up to 20 mm distance.

Similarly, the reader antenna is also tested for reading multiple tags positioned in various orientations above 10 mm foam. Additionally, the reader antenna can able to detect the tag blood bags or intravenous (IV) solution bags^3^ in order to avoid human or handwritten verification errors. Similarly, this reader antenna can detect low-cost ticket rolls with high precision and accuracy. The experimental findings demonstrate complete tag reading up to 20 mm above the antenna surface and further indicate that no tags are read outside the antenna surface, which is very useful for most near-field applications in order to prevent tag’s misreadings.

**Figure 15:**
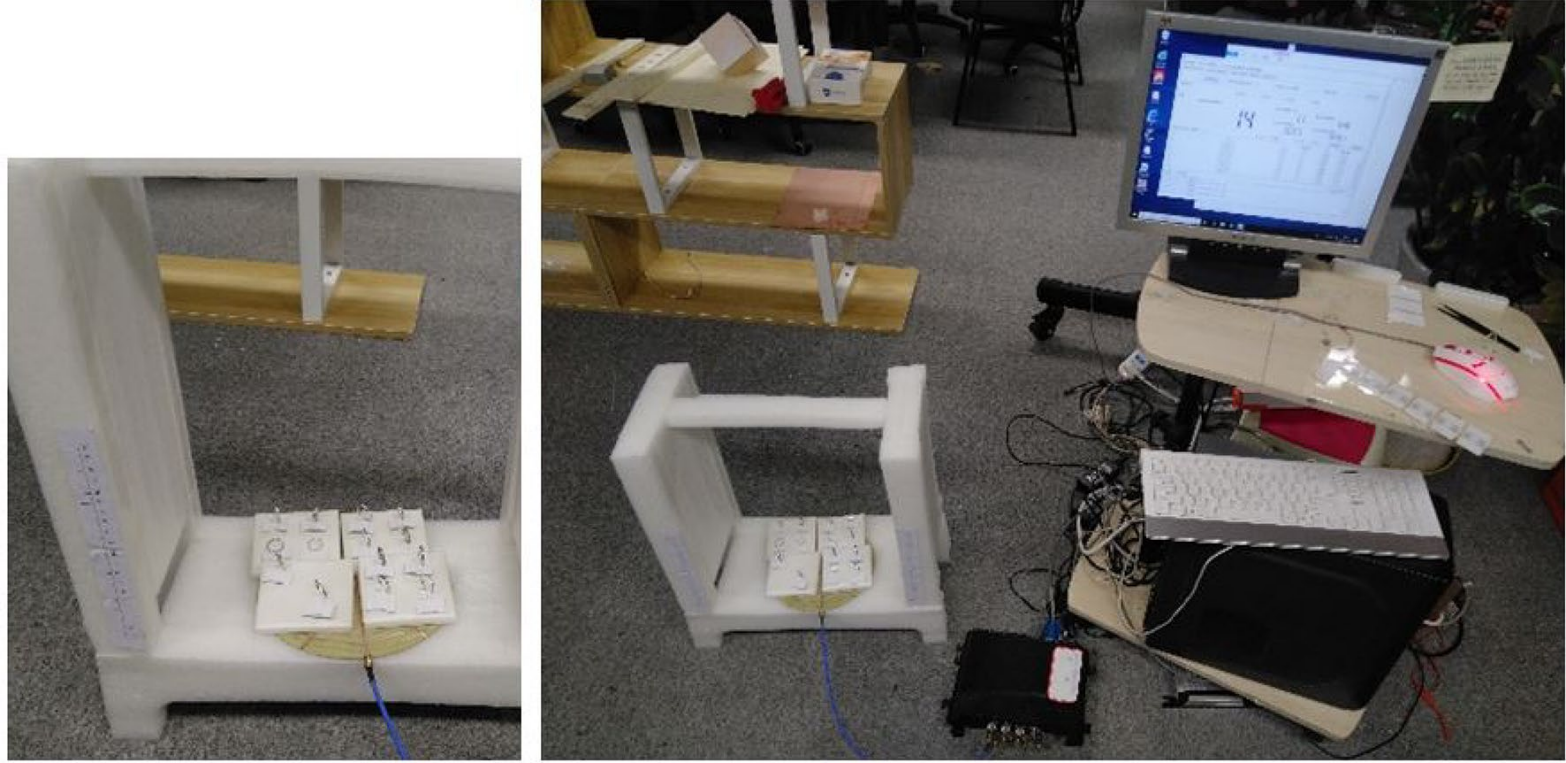
Experimental testing of proposed antenna design in reading tagged jewelry items in all orientations.

Furthermore, the tagged jewelry items put on cardboard boxes covered in silk cloths were tested to prove the effectiveness of the proposed near-field antenna for expensive item tagging as depicted in Fig. 15. The experimental setup shows this reader antenna successfully detects all tagged jewelry items with 100 % read accuracy.

## Conclusions

In this paper, a spider web-shaped ultra-high frequency (UHF) RFID reader antenna-based system for the Internet of Things (IoT) and healthcare applications is proposed. The reader antenna is based on eight concentric decagons of various sizes that are connected to the ground using 50 resistors, with the exception of the end that is left open for a feeding port. The reader antenna design has wideband properties be-because it covers the entire UHF RFID band (860–960 MHz) and has fairly strong and uniform electric field characteristics. In order to avoid other tags being misread in the majority of nearfield applications, it also has low gain characteristics. Further, the orientation sensitivity problems with low-cost linearly polarized tag antennas are addressed by the symmetric current distribution throughout the structure in this design. The measurement results demonstrate that tags can read expensive jewelry, tagged medicine pills, intervening solution, and blood bags positioned in different directions. In future, the array based on this reader antenna and small item-level tag can be used for counting and billing of expensive jewellery items in order to realize complete RFID based solution.

## Data Availability

The datasets used and/or analysed during the current study available from the corresponding author on reasonable request.
